# Improved Protein Semi-Synthesis Enables Biophysical Studies of Thioamide Destabilization of β-Sheet Interactions

**DOI:** 10.64898/2026.07.30.741859

**Published:** 2026-07-31

**Authors:** Evan S. K. Yanagawa, Kristen E. Fiore, Denver Y. Francis, Aiden Lesneski, Yanan Chang, Benjamin Roose, David Christianson, Kohei Sato, E. James Petersson

## Abstract

Thioamides are natural post-translational modifications of the peptide backbone and can be introduced synthetically to probe protein folding or functionalize peptides for translational applications. In this work, we demonstrate that thioamide-containing peptides with C-terminal thioesters can be efficiently generated using Knorr pyrazole activation and used in subsequent native chemical ligation reactions to generate thioamide containing proteins. We compare this method to acyl azide activation and find that both routes provide similar yields. We also investigate ultrasound-mediated desulfurization of the ligation site cysteine for potential advantages over chemical radical initiators. Scaling up our syntheses allows us to study thioamide perturbations to the β-sheet region of the B1 domain of protein G (GB1) as well as β-strand interactions in amyloid fibrils of the Parkinson’s disease protein α-synuclein. In both contexts, we observe dramatic destabilization of the β-sheet networks, manifested in decreased GB1 thermal stability and altered folding and slowed aggregation of α-synuclein. These findings illustrate the impact that a single atom substitution can have on cooperative hydrogen bonding networks and prompt future study of both systems.

## Full Text Availability

The license terms selected by the author(s) for this preprint version do not permit archiving in PMC. The full text is available from the preprint server.

